# Cytology and Human Papillomavirus Co-Test Results Preceding Incident High-Grade Cervical Intraepithelial Neoplasia

**DOI:** 10.1371/journal.pone.0118938

**Published:** 2015-03-20

**Authors:** Ina U. Park, Nicole Wojtal, Michael J. Silverberg, Heidi M. Bauer, Leo B. Hurley, M. Michele Manos

**Affiliations:** 1 Sexually Transmitted Disease Control Branch, Division of Communicable Disease Control, Center for Infectious Diseases, California Department of Public Health, Richmond, CA, United States of America; 2 University of California at Berkeley, Health and Medical Sciences, Berkeley, CA, United States of America; 3 Kaiser Permanente Northern California Division of Research, Oakland, CA, United States of America; State University of Maringá/Universidade Estadual de Maringá, BRAZIL

## Abstract

**Objective:**

High-risk HPV (hrHPV) and cytology co-testing is utilized for primary cervical cancer screening and for enhanced follow-up of women who are hrHPV-positive, cytology negative. However, data are lacking on the utility of this method to detect pre-cancer or cancer in community-based clinical practice. This study describes cytology and hrHPV results preceding high-grade cervical intraepithelial neoplasia, adenocarcinoma in situ, or cervical cancer (i.e., CIN2+) in an integrated health system employing routine co-testing among women aged 30 years and older.

**Methods:**

We conducted a cross-sectional analysis of adult female members of Kaiser Permanente Northern California (KPNC) with incident CIN2+ between July 2008 and June 2009. The primary outcome was the proportions of cytologic diagnoses and hrHPV co-test results preceding a diagnosis of CIN2+. Cervical cytology and hrHPV testing results were abstracted from electronic medical records.

**Results:**

Of 1283 CIN2+ cases among adult women, 880 (68.5%) were among women aged 30 years and older and 145/880 (16.5%, 95% CI 14.1–19.1) had only normal cytology during the 12 months prior to diagnosis. Furthermore, 133/880 (15.1%, 95% 12.9–17.7) were preceded by only normal cytology and persistent hrHPV infection (at least 2 positive hrHPV tests) during the 6–36 months preceding CIN2+ diagnosis.

**Conclusions:**

Incident CIN2+ is frequently preceded by normal cytology and persistent hrHPV infection among women aged 30 years and older; screening strategies that employ HPV testing and cytology may improve the detection of CIN2+ compared with cytology alone.

## Introduction

The key to cervical cancer prevention is the early detection and treatment of precancerous lesions. Though cytology has been the cornerstone of cervical cancer screening for more than 50 years, there are well-documented limitations with both the sensitivity and specificity of cervical cytology.[1, 2] Additionally, minimally abnormal cytologic diagnoses such as atypical squamous cells of undermined significance (ASC-US) are common in young women and if managed aggressively can lead to unnecessary procedures and adverse reproductive health outcomes. [3]

In addition to its established role in the triage of ASC-US cytology,[4, 5] high-risk human papillomavirus (hrHPV) testing with cytology (co-testing) has been demonstrated to facilitate early detection of CIN3+ among women aged 30 years and older. [6–8] Given the high negative predictive value of a negative cytology and hrHPV co-test, recent guidelines in the United States state that extending screening intervals to every 5 years is safe for this population. [2, 7, 9, 10] However, a substantial proportion of women with negative cytology are hrHPV-positive (4–15%). [11] Although enhanced surveillance with repeat co-testing is recommended for this population, it is unclear what proportion of women with an eventual diagnosis of CIN2+ demonstrate persistent cytology-negative, hrHPV positive results prior to diagnosis.

Since 2003, Kaiser Permanente Northern California (KPNC), a large, integrated health maintenance organization, adopted a policy of hrHPV co-testing in conjunction with Pap screening for women aged 30 years and older, providing a unique opportunity to examine outcomes of this screening strategy in a large patient population. An earlier published analysis of the KPNC population described cytology diagnoses preceding CIN2+ before the adoption of hrHPV co-testing among women. [12] The goal of the current study is to evaluate the distribution of cytologic diagnoses and hrHPV test results preceding a histologic diagnosis of CIN2+ in the period after the policy of hrHPV co-testing in women aged 30 years and older was adopted at KPNC. These observations from routine clinical practice may complement data from cohort studies and clinical trials to better understand the potential contribution of hrHPV co-testing in the detection of pre-cancerous cervical disease.

## Materials and Methods

### Study design, population and setting

We conducted a cross-sectional analysis with the aim of describing cytologic and hrHPV co-test results preceding incident, histologically confirmed cases of high-grade cervical intraepithelial neoplasia (CIN2, CIN 3, CIN 2/3), adenocarcinoma in situ (AIS), cervical cancer, or any combination thereof (CIN2+) among adult women at KPNC for the study period July 1, 2008 to June 30, 2009. The KPNC membership consists of 3.3 million members, corresponding to approximately 23% of the adult population of northern California, of which approximately 1.3 million are adult women. [13,14]

During the study period, KPNC clinical guidelines (based on national guidance) recommended cervical cancer screening for women aged 30 years of age or older with conventional slide-based cytology and simultaneous hrHPV co-testing using a separate swab in standard transport media (HC2 STM, Qiagen Corporation, Gaithersburg, MD). [15] For women in this age group who were hrHPV-positive with normal cytology, repeat cytology and hrHPV in 12 months was recommended. Colposcopic evaluation was recommended when repeat co-testing demonstrated either abnormal cytology or persistent hrHPV infection. For women under age 30, hrHPV co-testing was not recommended, however, reflex hrHPV testing of ASC-US cytology was routinely performed or could be ordered at the providers’ discretion.

### Case Definitions

CIN2+ cases were identified through a query of disease-specific Systematized Nomenclature of Medicine (SNOMED) classification codes in the KPNC electronic medical record (EMR). A natural language text search of pathology reports was performed to confirm the SNOMED diagnoses. Manual review of pathology reports occurred for cases not initially classified by the natural language text search and if indicated, SNOMED-identified diagnoses were reassigned based on the manual review. For patients with multiple CIN2+ diagnoses during the study period, only the earliest diagnosis (index CIN2+) was included.

To limit our analysis to likely incident CIN2+, we excluded patients with a diagnosis of CIN2+ within 24 months prior to the date of the index CIN2+ diagnosis. We required cases to have KPNC health plan membership for at least 22 of 24 months prior to the index CIN2+ diagnosis to ensure that sufficient historical data were available to permit exclusion of known prevalent CIN2+.

Data for each of the CIN2+ cases were obtained from the KPNC EMR, which includes comprehensive data on diagnoses, utilization, laboratory results, and pharmacy prescriptions. For all cases, we collected the following data: age at diagnosis, race/ethnicity, histologic diagnosis, up to three cytology test results and up to three hrHPV test results from the 12 months preceding the index CIN2+ diagnosis. Cytology diagnoses were reported according to the 2001 Bethesda System: ASC-US, low-grade squamous intraepithelial lesion (LSIL), high-grade squamous intraepithelial lesion (HSIL), atypical squamous cells—cannot exclude HSIL (ASC-H), atypical glandular cells (AGC), adenocarcinoma in situ (AIS), and squamous cell carcinoma (SqCA). [16] Cytology was classified by programmatic text search of electronic pathology reports (searching for Bethesda terms) with supplemental manual review of reports the program could not classify. Cytology was performed separately from hrHPV testing, so KPNC cytologists were not routinely aware of hrHPV test results when evaluating cytology results. hrHPV status was determined using the Hybrid Capture 2 hrHPV DNA assay (HC2, Qiagen Corporation, Gaithersburg, MD).

The cytology and hrHPV tests performed prior to the index CIN2+ diagnosis (within the period of 8 days to 12 months prior to diagnosis) were included in the analysis. A hrHPV DNA co-test was defined as any hrHPV test conducted within seven days before or after a particular cytology test. Finally, cytology and/or hrHPV tests performed concurrently or within 7 days of the index CIN 2+ biopsy date were disregarded, as these results did not trigger the decision to perform colposcopy/biopsy.

For CIN2+ cases preceded by normal cytology, all hrHPV test results in the 36 months prior to diagnosis were collected. These data were used to determine the proportion of women with normal cytology who had persistent positive hrHPV tests preceding the CIN2+ diagnosis. HPV persistence was defined as 2 or more positive hrHPV tests occurring 6–36 months prior to the date of the index CIN2+ diagnosis.

### Data Analysis

The following statistics were computed: 1) distribution of cytologic diagnoses preceding the index CIN2+ histologic diagnosis, 2) proportion of women who received a hrHPV co-test by cytologic diagnosis categories and 3) proportion of women with a positive hrHPV co-test by cytologic diagnosis categories. All results were stratified by age (18–29 years, ≥30 years) for consistency with national hrHPV co-testing guidelines. The Mantel-Haenszel chi-square test was used to compare proportions of cytology diagnoses between age strata. A separate analysis with women aged 40 years and older was conducted for comparison with results from a previous study in this population, which only stratified women as age <40 years or 40 years and older. [12] Data analyses were conducted using STATA version 10 (StataCorp, College Station, TX).

### Human Subjects

This study was approved by the Institutional Review Boards of the Kaiser Foundation Research Institute and the University of California at Berkeley, and included a waiver of informed consent.

## Results

We initially identified 2,215 adult women with histologically diagnosed CIN2+ during the study period. Of these, we excluded a total of 932 women (42% of 2215) resulting in a final study population of 1,283 women with incident CIN 2+. Study exclusions included: 667 women with fewer than 22 of 24 months membership prior to the CIN2+ diagnosis; 172 women with a CIN2+ diagnosis within the prior 24 months (prevalent cases); 58 women with no evidence of CIN2+ upon manual review of the pathology report; 23 women with no cytology or hrHPV testing at KPNC in the prior 12 months; and, 12 women with only a Pap test performed concurrently with histology.

Table 1 shows the characteristics of the study population. The mean age of women with CIN2+ was 36.7 years of age, and more than two-thirds of CIN2+ cases were among women aged 30 years and older. The population was racially/ethnically diverse: of the 83% of women with race ethnicity information, 48.3% were White/Caucasian, 12.1% were Black/African American, 21.7% were Hispanic/Latino, and 15.7% were Asian/Pacific Islander. Table 2 summarizes the distribution of cytology and hrHPV results preceding the CIN2+ histologic diagnosis in the population overall and stratified by age (18–29 years vs ≥30 years). A total of 151 women had normal cytology preceding index CIN2+, and diagnoses were distributed as follows: 69 (45.7%) had CIN2, 28 (18.5%) had CIN2/3, 53 (35.1%) had CIN3, and 1 (0.7%) had AIS. For women with abnormal cytology preceding CIN2+, no significant differences were observed in the proportions of abnormal cytology by age including ASC-US (p = 0.19), ASC-H (p = 0.49), AGC/AIS (p = 0.45) or HSIL (p = 0.53). hrHPV co-test positivity among women aged 30 years and older was greater than 90% for nearly all cytologic groups. The only exception was AGC, where hrHPV co-test positivity was less than 90%; however there were few women (n = 21) with this cytologic diagnosis.

**Table 1:** Demographic and Clinical Characteristics of Women with CIN2+ diagnosed between July 2008-June 2009 at Kaiser Permanente Northern California (n = 1,283).

Characteristic	N (%)
Age (range: 18–84 years)	
Mean (±SD)	36.7 (±11.8) years
Age Group	
18–29 years old	403 (31.4)
30–84 years old	880 (68.6)
Race/Ethnicity (N = 1069)	
Asian/Pacific Islander	168 (15.7)
Black/African-American	130 (12.1)
White/Caucasian	517 (48.3)
Hispanic/Latino	233 (21.7)
Mixed Race	15 (1.4)
Native American/Alaska Native	7 (0.7)
Histologic diagnoses	
CIN 2	527 (41.1)
CIN 2/3	471 (36.7)
CIN 3	256 (19.9)
AIS	29 (2.2)
***Women Age 30+ only (n = 880)***	
hrHPV co-test performed (±7 days of cytology)	850 (96.6)
Number of hrHPV co-tests in 12 months prior to CIN2+ diagnosis	
One	815 (92.6)
Two	65 (7.4)

SD (standard deviation); hrHPV (High-risk HPV).

**Table 2:** Distribution of cytology and high-risk HPV test results preceding CIN2+ diagnoses at Kaiser Permanente Northern California, July 1, 2008 to June 30, 2009 (n = 1,283).

Cytology category	Age 18–29 years			Age ≥ 30 years			All ages (range 18–84)	
	n	% of CIN2+ cases (95% CI)*	% hrHPV+ (n+/n tested)	n	% of CIN2+ cases (95% CI)*	% hrHPV+ (n+/n tested)	n	% of CIN2+ cases (95% CI)*
Normal	6	1.5	100	145	16.5	97.9	151	11.8
			(5/5)		(14.1–19.1)	(142/145)		(10.1–13.7)
ASC-US	132	32.8	99.2	256	29.1	99.2	388	30.2
		(28.2–37.6)	(127/128)		(26.1–32.2)	(247/249)		(27.8–32.9)
ASC-H	56	13.9	97.9	110	12.5	91.7	166	12.9
		(10.8–17.8)	(47/48)		(10.4–14.9)	(99/108)		(11.2–14.9)
AGC	5	1.2	66.7	16	1.8	84.6	21	1.6
			(2/3)		(1.1–3.0)	(11/13)		(1.0–2.5)
LSIL	120	29.8	87.5	152	17.3	93.8	272	21.2
		(25.4–34.5)	(21/24)		(14.9–20.0)	(136/145)		(19.0–23.6)
HSIL	84	20.8	100	197	22.4	93.0	281	21.9
		(17.1–25.2)	(23/23)		(19.7–25.3)	(174/187)		(19.7–24.3)
AIS	-	-	-	1	0.1	100	1	0.1
						(1/1)		
SqCA	-	-	-	3	0.3	100	3	0.2
						(2/2)		
Total	403	100	97.4	880	100	97.1	1,283	100
			(225/231)			(812/850)		

CIN2+ (cervical intraepithelial neoplasia grade 2 or worse); hrHPV (high-risk human papillomavirus); ASC-US (atypical squamous cells of undetermined significance); ASC-H (atypical squamous cells—cannot exclude HSIL); AGC (atypical glandular cells); LSIL (low-grade squamous intraepithelial lesion); HSIL (high-grade squamous intraepithelial lesion); AIS (adenocarcinoma in situ); SqCA (squamous cell cancer); 95% CI (95% confidence interval).

* not listed if n <10.

Among CIN2+ cases in women aged 30 years and older (n = 880), 144/880 (16.5%, 95% CI 14.1–19.1) were preceded by only normal cytology in the 12 months prior to diagnosis; none were cancer and 1 was a case of AIS. Similar results were observed for CIN3+ cases, 28/205 (13.7%) were preceded by only normal cytology (p = 0.34). Nearly all CIN2+ cases had a positive hrHPV co-test result (142/144, 98.6%); reasons for colposcopy of patients with negative hrHPV and normal cytology were not known. Furthermore, the majority (135/144, 93.8%) also had at least 2 hrHPV test results in the period 6–36 months prior to CIN2+ diagnosis, with a mean time between hrHPV tests of 14.6 months (range 6.2–32.7 months). Nearly all cases with multiple hrHPV results (133/135, 98.5%) were persistently hrHPV positive. Accounting for all available hrHPV testing data up to 36 months prior to CIN2+ diagnosis, 15.1% (133/880) of CIN2+ diagnoses in women aged 30 years and older were preceded by normal cytology but persistent hrHPV infection.

**Fig 1:**
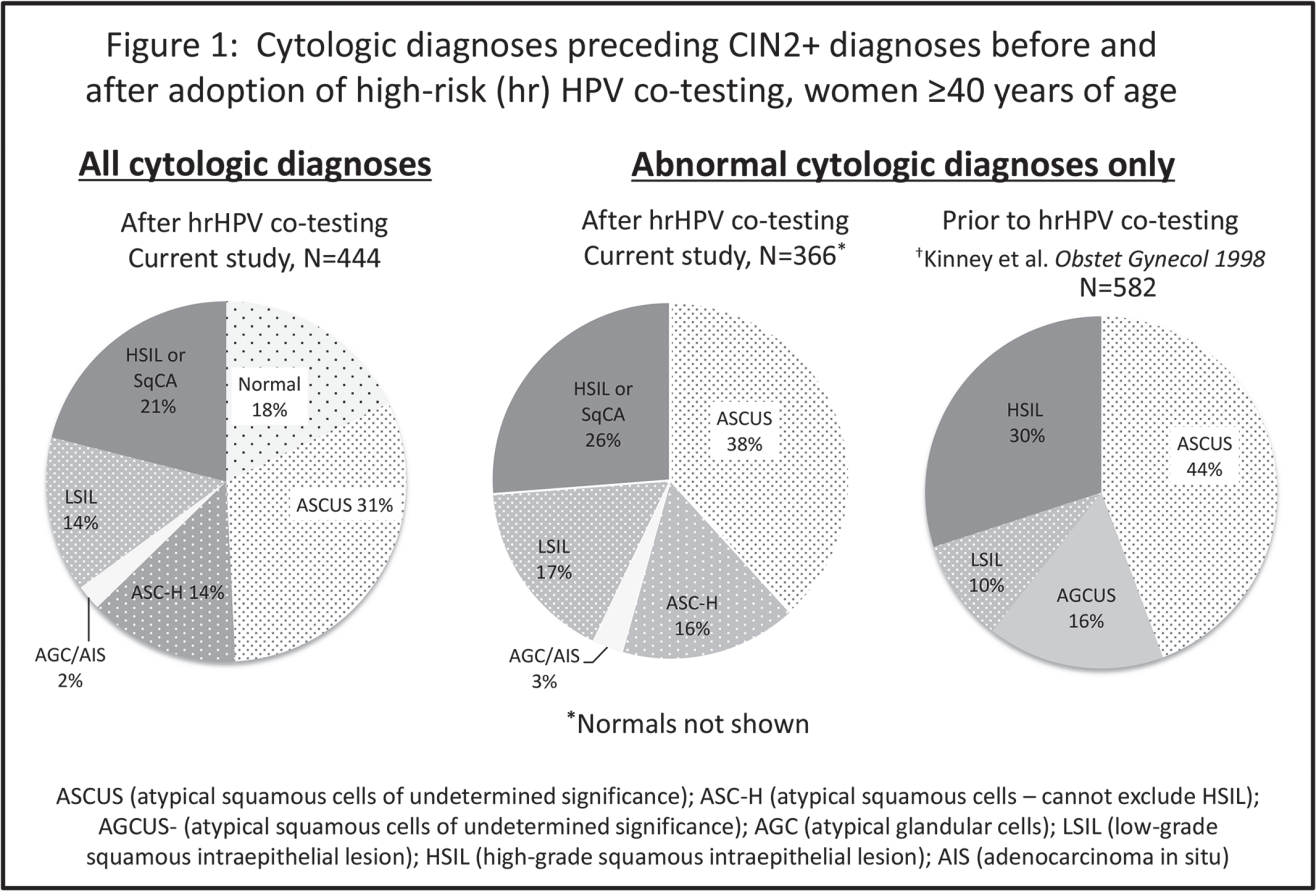
Cytologic diagnoses preceding CIN2+ diagnoses before and after adoption of high-risk (hr) HPV co-testing, women ≥40 years of age. ASC-US was most common cytologic diagnosis during both time periods, followed by HSIL.

Among women aged 40 years and older, Fig. 1 demonstrates the distribution of cytologic diagnoses preceding CIN2+ compared to the prior study conducted within the KPNC membership by Kinney et al. [12] Both cytology nomenclature and screening practices have changed in the time between the two studies (1996 and 2009); cytologic categories reported in the current study but not reported in Kinney et al include: normal, AGC/AIS, and ASC-H. Normal cytology preceded 18% of CIN2+ in women aged 40 years and older in the current study. When comparing abnormal cytologic results alone between the current and prior study, an ASC-US diagnosis was similarly common immediately prior to an index CIN2+ (38% vs 44%, p = 0.06). Proportions of LSIL and HSIL were also similar. In contrast, Kinney et al found 16% of women aged 40 years and older had AG-CUS cytology preceding CIN2+ compared with only 2% with AGC/AIS cytology in the current analysis (p<0.0001), while 16% of current index CIN2+ cases were preceded by ASC-H, which did not exist as a diagnostic category at the time of the prior study.

## Discussion/Conclusions

Our analysis illustrates the key role of hrHPV co-testing in identifying CIN2+ among women 30 years of age and older with normal cytology. Our findings suggest that if women aged 30 years and older had not been co-tested for hrHPV as part of KPNC’s cervical cancer screening protocol (i.e., they had only received cytology), 15.1% of 880 CIN2+ cases in this age group may have gone undetected. For these cases, hrHPV co-testing results prompted a referral for colposcopy/biopsy that would not have occurred using algorithms that rely only on abnormal cytology. Nearly half of CIN2+ cases preceded by normal cytology were CIN2; which has been estimated to regress in up to 40% of women in the ASCUS-LSIL Triage Study (ALTS). [17] However, the mean age in ALTS was 24.8 years (median 23 years) and CIN2 regression is less likely in older women (including those in the age range indicated for co-testing). Though national management guidelines recommend immediate treatment for CIN2 in older women (age >25 years), [18] debate still exists as to whether detection of CIN2 with screening is a desired outcome.

In comparing current findings from those of Kinney et al among women aged 40 years and older in the KPNC population, proportions of ASC-US, LSIL and HSIL cytology prior to index CIN2+ were similarly distributed in both studies. However, there were notable differences in distribution of glandular cytologic abnormalities; the reason for this observed is unclear. The current study includes the ASC-H category which did not exist previously, it is unknown if some of these women had co-existing mild glandular abnormalities that would have previously been classified as AG-CUS. Cytology interpretation practices and practitioners may have differed between the two studies or perhaps there was an influence of hrHPV co-testing on identifying patients early who might later present with AGC/AIS cytology.

Multiple European trials of cervical cancer screening have compared performance of cytology alone to cytology plus hrHPV co-testing, and, similar to the current study, have also found a substantial proportion of women with CIN2+ preceded by normal cytology and hrHPV positivity (7.3%-25.3%).[19–23] The age ranges of the study populations and methods of hrHPV testing used in these trials varied from clinical practice in the United States (US), which may account for the wide range of hrHPV positivity observed. KPNC practice guidelines are similar to those of the American Society for Colposcopy and Cervical Pathology, and thus the proportion of cytology- negative, hrHPV-positive women observed in our study (16.5%) is likely more reflective of other U.S insured populations than estimates from European trials.

A similar study to ours by Zhao et al in a university hospital setting found a high proportion (61%) of women with CIN2/3 had at least 1 normal cytology in the 4 month-3 year period before index diagnosis, however they did not report any CIN2/3 cases with normal cytology in the 4 months immediately prior to diagnosis. [24] Given that only 28.6% patients had hrHPV testing results associated with the index CIN2/3 diagnosis, this likely reflects less hrHPV co-testing in this practice compared to the routine hrHPV co-testing performed in our population.

There are a number of limitations to our study. Though KPNC membership is similar to the population of Northern California (including Medicaid patients) with respect to race/ethnicity, it under-represents the very poor and over-represents employed populations. [14] To ensure we were capturing incident CIN2+ cases, we excluded a large proportion of cases (42%) from analysis (primarily due to short membership); however it is unlikely that length of membership would have a large influence on screening results. KPNC utilized conventional cytology for cervical screening during the study period; however, clinical trials and multiple meta-analyses have shown equivalent or nearly equivalent performance of liquid-based and conventional cytology for identifying high-grade cervical neoplasia.[25–28] We attempted to exclude all women with prevalent CIN2+ by excluding those with a prior diagnosis of CIN2+ in past 24 months, as well as any who were members for fewer than 22 of the 24 prior months. However, some cases that met these eligibility criteria may have had recurrent or prevalent disease that was initially diagnosed more than 24 months prior. Additionally, the identification of CIN2+ via SNOMED diagnosis codes could have missed some cases of CIN2+; however any misclassification would not likely differ according to age, cytologic diagnosis, or other factors.

Finally, diagnosis of CIN2+ relies on visualization of lesion during colposcopy; data on adequacy of colposcopy procedures among women with positive co-tests were not available.

Although hrHPV co-testing may be an effective method to improve cervical disease detection, it is important to consider potential harms of co-testing, such as patient anxiety and additional medical procedures including overtreatment that might occur as a result of only transiently positive hrHPV test results. [29–31] These harms will be exacerbated by improper use of hrHPV testing, including co-testing young women or co-testing more frequently than currently recommended. Additionally, debate exists over how best to utilize hrHPV testing and whether co-testing or primary hrHPV testing alone should replace cytology. Recently released U.S. consensus guidelines have recommended cytology with hrHPV co-testing with 5 year screening intervals over cytology alone in women 30 years of age and older, [10] and the US Food and Drug Administration recently approved primary hrHPV testing alone as a screening methodology in women aged 25 years and older. [32] Furthermore, the landscape of screening recommendations and optimal screening methodology/frequency will evolve as HPV vaccination becomes more widely adopted.

In conclusion, co-testing for hrHPV appears to contribute substantially to the identification of high grade cervical disease in women 30 years of age and older. Specifically, in this study, hrHPV co-testing identified an additional 15% of CIN2+ cases compared with cytology alone among women 30 years of age and older. Further investigation is needed to determine how hrHPV testing can be used most effectively in cervical cancer screening protocols to maximize disease detection, avoid unnecessary diagnostic procedures, and reduce loss to follow-up.
